# Augmentation or erosion? Artificial intelligence and the transformation of clinical reasoning: a critical narrative review

**DOI:** 10.3389/fdgth.2026.1949323

**Published:** 2026-10-21

**Authors:** José Daniel Sánchez

**Affiliations:** Facultad de Ciencias de la Salud y Bienestar Humano, Universidad Tecnológica Indoamérica, Quito, Ecuador

**Keywords:** artificial intelligence, automation bias, clinical decision support systems, clinical reasoning, deskilling, diagnostic error, health equity, large language models

## Abstract

**Background:**

Artificial intelligence (AI) systems are used to support diagnosis, but a model’s accuracy does not establish that clinicians reason more accurately when they use it. This review appraises how AI decision support and large language models affect physicians’ diagnostic performance and the risks of relying on them.

**Methods:**

This critical narrative review follows the Scale for the Assessment of Narrative Review Articles. Scopus, PubMed/MEDLINE, SciELO and LILACS were searched in July and August 2026 in English, Spanish and Portuguese. Eligible studies, published from 2015 onward, compared AI-assisted with unassisted clinicians, measured reliance on AI or unassisted performance after exposure to it, or externally validated deployed models. A single author selected sources purposively. Nine primary studies were appraised with an adapted Critical Appraisal Skills Programme framework.

**Results:**

In randomized trials of perceptual tasks, AI support improved detection. In the MASAI mammography screening trial, cancer detection rose from 5.0 to 6.4 per 1,000 women, reading workload fell by 44%, and the interval-cancer rate was non-inferior. In colonoscopy, computer-aided detection raised the adenoma detection rate from 40.4% to 54.8%. In the only randomized trial of open-ended diagnostic reasoning, access to GPT-4 did not improve physicians’ scores (76% vs. 74%; P=.60), although the model alone scored 92%. Evidence of harm came from smaller or non-randomized studies: reader accuracy fell sharply when AI suggestions were incorrect or the AI missed a cancer; physicians trained in AI literacy were still misled by erroneous model advice; unassisted adenoma detection fell from 28.4% to 22.4% after routine AI use in one pre–post study; and a deployed sepsis model discriminated poorly on external validation (area under the curve 0.63). No eligible study from Latin America was identified.

**Conclusions:**

In the studies reviewed, AI assistance improved clinician performance in bounded detection tasks but not in open-ended diagnostic reasoning, where evidence is limited to one vignette-based trial. The evidence that reliance on AI can impair performance comes from single or design-limited studies and does not establish how large or lasting such effects are. Benefit therefore cannot be inferred from model accuracy alone; assisted and unassisted clinician performance should be measured directly.

## 1 Introduction

Clinical reasoning is the cognitive core of medical practice. Through it, clinicians elicit the patient’s narrative, interpret physical and laboratory findings, rank competing diagnoses and plan treatment under uncertainty (1, 2). It is also a recognized source of preventable harm. Combining data from three large observational cohorts, Singh and colleagues estimated an outpatient diagnostic error rate of 5.08%, affecting approximately 12 million adults in the United States each year (3). Newman-Toker and colleagues later calculated that about 795,000 people in the United States die or are permanently disabled each year as a result of diagnostic error, and that fifteen conditions account for more than half of these serious harms (4).

Because so much of this harm is concentrated in a small number of conditions, algorithmic assistance is an attractive remedy: a system that raised clinicians’ suspicion of high-morbidity diseases at the right moment could, in principle, prevent many missed diagnoses (5). This expectation depends on an assumption that is seldom examined, namely that a model’s accuracy transfers to the clinician who uses it. Conventional diagnostic instruments, such as laboratory analyzers or physiological monitors, supply discrete data that the clinician interprets. Contemporary AI systems and clinical decision support systems (CDSS) instead supply interpretations, diagnostic suggestions and risk estimates, which enter the clinician’s deliberation directly. What needs to be evaluated is therefore the clinician and the system working together in clinical workflows, and a model’s performance on a curated benchmark is not a substitute for that evaluation.

This review takes the clinician working with the system as its unit of analysis. Its first aim is to establish what randomized and prospective studies show about AI-assisted diagnosis, in perceptual tasks and in open-ended reasoning. Its second aim is to appraise the evidence on four ways in which reliance on AI may harm diagnostic performance: automation bias, loss of unassisted skill, undetected model failure and demographic disparity. On the basis of these findings, the Discussion considers whether explainable AI and mandatory human oversight, the safeguards most often proposed, address these risks, and what the evidence implies for governance, education and health systems in Latin America, where algorithmic tools are usually imported without local clinical evaluation.

## 2 Methods

### 2.1 Design and reporting framework

This critical narrative review is reported according to the Scale for the Assessment of Narrative Review Articles (SANRA) (6), which assesses six dimensions: justification of the topic’s importance, statement of concrete aims, description of the literature search, referencing, scientific reasoning, and presentation of relevant data. An item-by-item adherence statement is provided in Supplementary Table S1. The aim was to interpret a heterogeneous body of evidence, not to estimate a pooled effect, and a systematic review design was therefore not adopted: no meta-analysis was performed, screening was not duplicated, and no formal risk-of-bias tool was applied across the retrieved records.

### 2.2 Information sources and search strategy

Four databases were searched: Scopus, PubMed/MEDLINE, SciELO (Scientific Electronic Library Online) and LILACS (Latin American and Caribbean Health Sciences Literature). The search was first run on 26 July 2026 and updated on 20 August 2026, with identical date limits in every database. Queries combined controlled vocabulary, including MeSH terms where available, with free-text keywords along three conceptual axes. The first described the technology (artificial intelligence, machine learning, deep learning, large language models, generative AI and clinical decision support systems). The second described the cognitive and clinical process (clinical reasoning, diagnostic error, diagnostic accuracy, differential diagnosis, automation bias, deskilling and human–AI interaction). The third, used in the regional stream, described geographical and equity scope (Latin America, South America, Ecuador, Andean, developing countries and health equity).

In SciELO and LILACS the strings were translated into Spanish and Portuguese, for example *“inteligencia artificial”* AND (*“razonamiento clínico”* OR *“apoyo a la toma de decisiones clínicas”*) and *“inteligência artificial”* AND *“raciocínio clínico”*. A PubMed search restricted by Latin American geographical terms was run as a separate regional stream. The database searches were complemented by hand-searching the reference lists of retrieved studies and reviews and by citation chaining to the primary source of every quantitative claim. The complete syntax for each database, with execution dates, is reproduced in Supplementary Table S2a so that the search can be repeated independently.

### 2.3 Eligibility criteria

The inclusion criteria were defined in terms of population, exposure, comparator, outcome, design and study period. The population comprised practicing physicians and physicians in training who made diagnostic, screening or detection decisions, in any specialty and care setting; readers interpreting images within screening programs were included on the same basis. The exposure was the use, during the diagnostic task, of an AI system that generated a diagnostic output, whether a machine-learning CDSS, a computer-aided detection tool or a large language model (LLM). Eligible studies compared assisted performance with unassisted performance or with standard practice, either between groups or within the same clinicians, and reported a clinician-level or patient-level outcome such as diagnostic accuracy, detection rate, a validated reasoning score, time to treatment, or a measure of reliance on the system.

Randomized controlled trials (RCTs), non-randomized comparative trials, prospective observational studies and controlled reader experiments were eligible. Studies of unassisted performance after exposure to AI, and independent external validations of models already deployed in clinical care, were also included, because these designs show how systems behave in use and not only during development. For contemporary empirical evidence, the study period extended from 2015 to the date of the final search; no date limit was applied to foundational cognitive, methodological and normative literature. Records in English, Spanish and Portuguese, the languages of the search strings, were assessed. Only peer-reviewed publications whose bibliographic record could be confirmed with the publisher or a bibliographic index were accepted as sources of primary data.

Two further categories of source were used for purposes other than evidence. Systematic and scoping reviews published between 2024 and 2026 on clinical reasoning and generative AI served to identify primary studies and are cited only as signposts. Statutory, intergovernmental and appraisal instruments are cited as normative or methodological sources.

Studies were excluded when they evaluated algorithmic performance only on retrospective benchmark datasets without clinician involvement, because such studies cannot inform the interaction that is the subject of this review. Commentaries, editorials, preprints and commercial white papers were not accepted as sources of primary data, nor were unverified consultancy metrics or promotional claims. Duplicate reports of an included study were excluded, except when separate protocol-defined analyses of the same trial reported different outcomes; in that case each report is cited for the outcome it reports, as with the three MASAI publications described in Section 4.1.1.

### 2.4 Study selection

Selection was purposive, not exhaustive. The author screened records against the criteria above and assessed in full text any record whose eligibility was uncertain at the title and abstract stage. When several sources supported the same point, the study with the strongest design and the most directly relevant outcome was retained.

Screening was performed by a single reviewer, so selection errors cannot be excluded, although full-text assessment of all uncertain records reduces this risk. No record-level screening log was kept prospectively. The review therefore reports neither the numbers of records identified, screened and excluded nor a flow diagram, because figures reconstructed afterwards could not be audited and would overstate the reproducibility of the process. Two elements can be audited and are provided: the executable search protocol (Supplementary Table S2a) and a classification of every cited source by its route of identification, whether database search, citation chaining, or foundational, methodological or normative literature outside the search (Supplementary Table S2b). Consequently, the review makes no quantitative claim about the size or completeness of the evidence base in any region, and statements about the content of the literature refer to what this search retrieved.

Quantitative values encountered in secondary reviews (2, 7–14) were traced to their primary publications before use, and the primary source is cited for each value. Every reference was checked against the publisher’s record, PubMed or the official legal text.

### 2.5 Critical appraisal of anchor studies

The nine primary studies on which the review’s principal findings rest were appraised with a framework adapted from the Critical Appraisal Skills Programme (CASP) checklists for trials, cohort studies and diagnostic studies (15). They comprise the MASAI mammography screening trial (16–18), a trial of computer-aided detection in colonoscopy (19), a trial of LLM-assisted diagnostic reasoning (20), an automation-bias experiment in mammography (21), an eye-tracking reader study (22), a trial of automation bias among physicians trained in AI literacy (23), a study of skill retention in colonoscopy (24), an external validation of a sepsis model (25) and an audit of algorithmic equity (26). Each study was assessed for clarity of the research question, appropriateness of the design, representativeness of the sample, validity of outcome measurement, handling of confounding and generalizability. The completed matrices are provided in Supplementary Table S3 and summarized in Table 3.

### 2.6 Classification of evidentiary strength

To keep interpretation separate from findings, each principal claim was assigned one of four levels. A claim was classed as *demonstrated* [D] when it was supported by at least one randomized or prospective study with an appropriate comparator and a patient- or task-level outcome; the label refers to the setting studied and does not imply generalization beyond it. A *signal* [S] rested on a single study or on designs open to confounding, such as pre–post comparisons, simulated reading experiments or trials lacking the comparator needed for the inference, and is therefore hypothesis-generating. A *hypothesis* [H] is mechanistically plausible but has not been tested directly in the literature identified. A *normative proposal* [N] is a recommendation advanced by the author and not an empirical finding. The classification of each principal claim is reported in Table 2 and mapped to its sources in Supplementary Table S4; the bracketed labels are also used in Table 1 and Figure 1. In the running text, the status of a claim is stated in words.

**Table 1:** Conceptual comparison of unassisted clinical reasoning and algorithmic decision support.

Dimension	Unassisted clinical reasoning	Algorithmic decision support
Knowledge base	Compiled illness scripts; experiential instance memory (29)	Statistical pattern associations; no experiential or physical embodiment [H]
Simultaneous hypotheses	Bounded working memory capacity (≈4 chunks) (31)	High-dimensional joint probability space; constrained by training distribution and architecture [H]
Temporal stability	Degrades with fatigue, interruption and high caseload (28)	Numerically invariant output for a fixed input; susceptible to silent drift under epidemiological shift (25)
Characteristic errors	Cognitive heuristics: anchoring, availability, premature closure (1, 30)	Dataset bias, shortcut learning, proxy-label misspecification (26)
Error detectability	Peer review and clinical consensus can identify individual reasoning lapses [H]	Explicit contradictions are recognizable at the bedside, but cohort-level calibration decay was not detected in routine use in the case examined (25)
Contextual integration	Can elicit implicit social, functional and goals-of-care preferences [H]	Operates on digitized and tokenized parameters [H]
Accountability	Attributable to an identified licensed clinician (32, 33)	Diffused across developers, deployers and operating users (32, 34)

Rows with citations restate published findings; rows labeled [H] are author-advanced propositions offered as an organizing scaffold and have not been established by direct comparative study.

**Figure 1:**
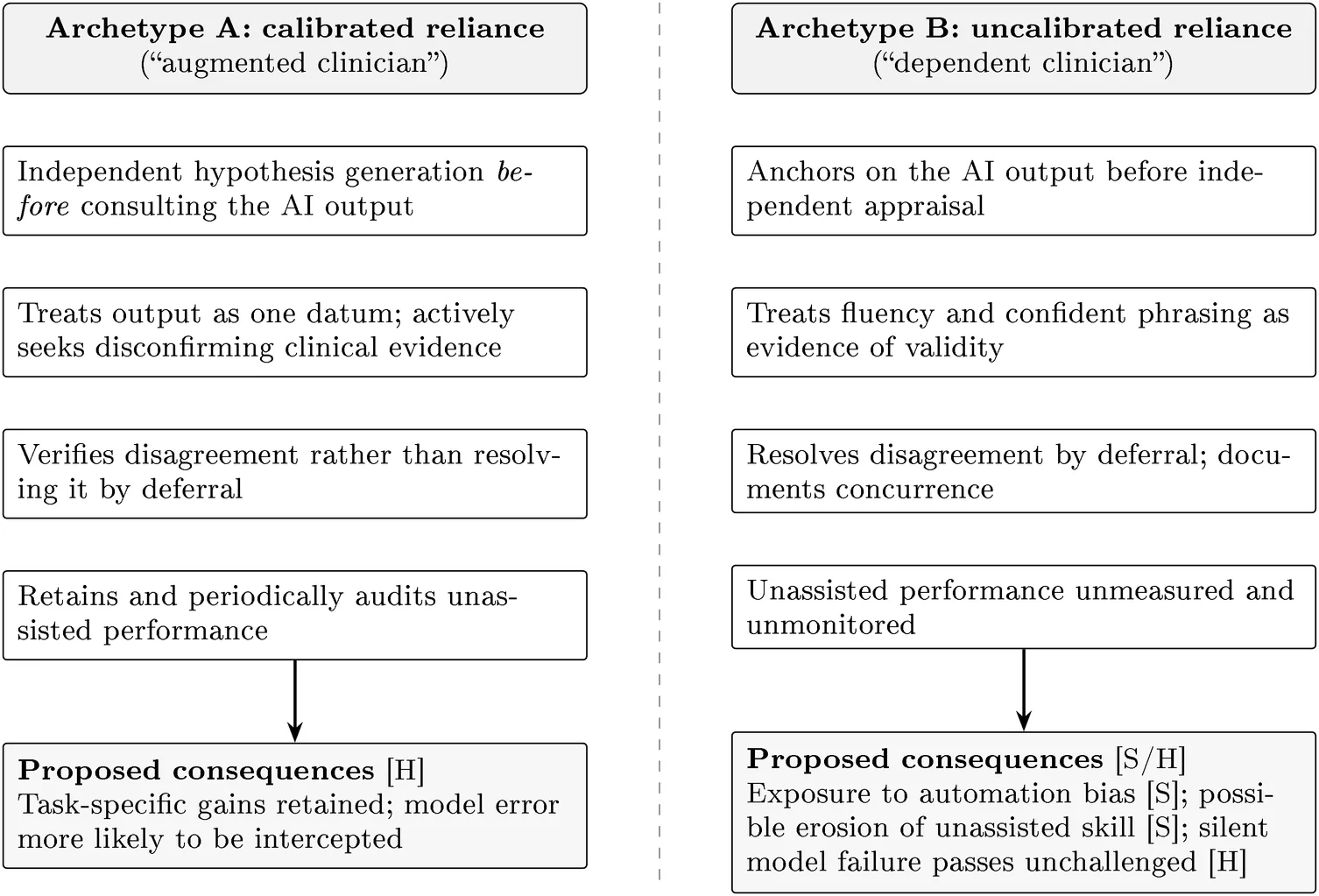
Conceptual framework of clinician–AI reliance archetypes. This figure is a conceptual framework, not a data display. The two columns are poles along a continuum of reliance calibration, not empirically observed clinician categories, and no causal effect of archetype membership on patient outcomes has been demonstrated. Labels follow the evidentiary classification of Section 2.6: [S] denotes signals from single or design-limited studies, [H] denotes author-advanced hypotheses.

## 3 Conceptual framework

### 3.1 Dual-process reasoning and its limits

Dual-process theory is the dominant model of diagnostic reasoning. It describes a fast, intuitive mode based on pattern recognition (System 1) interacting with a slower, analytical and hypothetico-deductive mode (System 2) (1, 27, 28). Expert clinicians rely on neither mode exclusively; expertise consists in moving between rapid recognition and deliberate checking, guided by awareness of one’s own reasoning. Much of the knowledge that makes rapid recognition possible is stored as illness scripts, compiled schemas that link predisposing factors, pathophysiology and clinical manifestations (29).

A common reading of this model attributes diagnostic error mainly to System 1 shortcuts and assumes that slower deliberation would prevent it. The empirical literature does not support that reading: cognitive biases and knowledge deficits contribute to diagnostic failure to a comparable degree, and interventions aimed only at debiasing have produced inconsistent benefit (30). This matters for the design of decision support. If many errors arise from gaps in knowledge or from difficulty discriminating between competing hypotheses, and not from insufficient deliberation, then AI support would be expected to help most when it improves access to relevant knowledge and sharpens discrimination without adding to the clinician’s cognitive load. That expectation has not been tested directly in the studies identified.

### 3.2 Human constraints and algorithmic properties

Human reasoning also operates within well-characterized limits. Working memory holds about four chunks of information (31), which restricts how many competing hypotheses a clinician can weigh under time pressure, and vigilance declines with fatigue, interruption and heavy caseloads (28). Computational systems do not tire and can operate across high-dimensional probability distributions, but they fail in other ways: they are sensitive to shifts in the data they receive, they can rely on spurious shortcuts learned from training data, and they lack experiential context.

Table 1 sets these properties side by side along the dimensions most relevant to understanding failure. It is a conceptual framework and not a summary of head-to-head comparisons: cited rows restate published findings, and uncited rows are the author’s propositions, labeled [H].

The comparison in Table 1 indicates that the two forms of reasoning fail in different ways, so that what matters in practice is how they combine. Figure 1 proposes two patterns of reliance that are used in the Discussion to interpret the findings. In calibrated reliance, the clinician forms hypotheses before consulting the system, treats its output as one item of evidence and verifies any disagreement; in uncalibrated reliance, the output anchors judgment and disagreement is resolved by deferring to the system. The two patterns are poles of a continuum, not observed categories of clinicians, and the arrows in the figure represent proposed relationships that have not been estimated.

## 4 Results

Findings are presented in the order of the two aims. The effect of AI assistance on diagnostic performance is reported first for perceptual tasks, in which the system flags findings in images or during procedures, and then for open-ended reasoning, in which the clinician must synthesize unstructured information into a differential diagnosis and a management plan. The evidence on harm follows, and the section closes with the regional search and a summary of evidentiary strength.

### 4.1 Perceptual tasks

The strongest evidence of benefit comes from breast screening and colonoscopy, both of which have been evaluated in randomized trials.

#### 4.1.1 Screening mammography

The Mammography Screening with Artificial Intelligence (MASAI) trial randomized women in the Swedish population-based screening program to AI-supported screen reading or to standard double reading. It has produced three protocol-defined analyses, which concern different populations and outcomes and are therefore described in turn.

The interim clinical safety analysis included the first 80,033 participants (16). Cancer detection was 6.1 per 1,000 screened (95% CI 5.4–6.9) with AI support and 5.1 per 1,000 (4.4–5.8) with standard reading, a ratio of 1.2 (95% CI 1.0–1.5; P=.052). The difference was not statistically significant at this stage, and the analysis was reported as evidence that detection had not declined. Recall rates were 2.2% and 2.0%, the false-positive rate was 1.5% in both arms, and screen-reading workload fell by 44.3%.

The analysis of the full randomized population, 105,934 participants after 19 exclusions, strengthened this result (17). Detection was 6.4 per 1,000 (95% CI 5.7–7.1) vs. 5.0 per 1,000 (4.4–5.6), a ratio of 1.29 (95% CI 1.09–1.51; P=.0021), corresponding to a relative increase of about 29%. The positive predictive value of recall was also higher with AI support (ratio 1.19, 95% CI 1.04–1.37; P=.012), and workload fell by 44.2%; this figure differs slightly from the interim estimate because it was computed in the larger population.

Higher detection is reassuring only if it is not offset by cancers that surface between screening rounds, and the completed trial addressed this question (18). Interval-cancer rates were 1.55 (95% CI 1.23–1.92) and 1.76 (1.42–2.15) per 1,000 participants, a non-inferior proportion ratio of 0.88 (95% CI 0.65–1.18; P=.41). Sensitivity was higher with AI support (80.5%, 95% CI 76.4–84.2, vs. 73.8%, 68.9–78.3; P=.031), and specificity was identical at 98.5%. Descriptively, interval cancers in the intervention arm were less often invasive (75 vs. 89), of stage T2 or higher (38 vs. 48), or of non-luminal A subtype (43 vs. 59). MASAI thus adds a downstream screening endpoint to the evidence on detection. Breast-cancer mortality and overdiagnosis were not endpoints of any of the three analyses, and the effect of AI-supported reading on them is unknown.

#### 4.1.2 Colonoscopy

Computer-aided detection (CADe) during colonoscopy has been evaluated against the adenoma detection rate (ADR), the principal quality indicator for colorectal cancer prevention. In a multicenter RCT of 685 patients, Repici and colleagues found that real-time CADe raised the ADR from 40.4% to 54.8% (relative risk 1.30, 95% CI 1.14–1.45) (19). The gain was concentrated in small adenomas, those of 5 mm or less (33.7% vs. 26.5%; RR 1.26, 95% CI 1.01–1.52) and those of 6–9 mm (10.6% vs. 5.8%; RR 1.78, 95% CI 1.09–2.86), and was independent of morphology and location. Wang and colleagues reported a similar effect in 1,058 patients, with the ADR rising from 20.3% to 29.1% (P<.001) (35). The absolute gain in the two trials, roughly 9 to 14 percentage points, largely reflects their different baseline ADRs. As with mammography, neither trial measured colorectal cancer incidence or mortality.

#### 4.1.3 Autonomous screening and acute triage

Two further trials concern autonomous screening and clinical workflow. In a pivotal prospective trial in 900 people with diabetes, an autonomous deep-learning system for diabetic retinopathy achieved 87.2% sensitivity and 90.7% specificity, meeting its pre-specified FDA endpoints without specialist over-reading at the point of care (36). In acute stroke care, a stepped-wedge cluster randomized trial in four comprehensive stroke centers found that automated detection of large-vessel occlusion on CT angiography, combined with secure group messaging, shortened the time to initiation of endovascular thrombectomy by about 11 min among 243 treated patients (37); the trial was powered for workflow times and not for functional outcome.

In all of these settings the task was bounded, the system produced a localized flag or an alert instead of a diagnostic conclusion, and the outcome could be measured at the point of care. Except with the autonomous retinopathy system, a clinician remained responsible for the final decision.

### 4.2 Open-ended diagnostic reasoning

Randomized evidence on whether generative AI helps physicians with complex, open-ended reasoning is scarcer and less favorable. The most rigorous test identified is the trial by Goh and colleagues, in which 50 attending and resident physicians in internal, family and emergency medicine were randomized to work through complex clinical vignettes with conventional digital resources alone or with those resources plus GPT-4 (20). Blinded expert raters scored performance with a validated diagnostic reasoning rubric.

Access to the LLM did not measurably improve physician performance. The median score was 76% (IQR 66–87) with the model and 74% (IQR 63–84) without it, an adjusted difference of 2 percentage points (95% CI −4 to 8; P=.60). The confidence interval excludes a large benefit but not a modest one, so the result is a null finding within a bounded interval and not evidence of equivalence. In a secondary analysis, GPT-4 working alone on the same vignettes achieved a median score of 92% (IQR 82–97), 16 percentage points higher than physicians using conventional resources (95% CI 2 to 30; P=.03).

The trial did not measure why the model’s advantage was not reproduced when physicians used it. The explanations proposed by its authors, which include ineffective prompting, anchoring on initial differentials and dismissal of correct suggestions that contradicted the physician’s first impression, were not tested and remain hypotheses (20).

Evaluations of generative models in medical education also indicate that their output requires expert review. When clinical experts assessed illness scripts generated by GPT-4 for 184 diseases, they judged 56.0% sufficient for teaching, 28.3% partially deficient and 15.8% inadequate (38). Reviews of CDSS for differential diagnosis in oral and maxillofacial radiology (7) and of agentic systems in experimental design (14) likewise describe utility confined to specific tasks.

### 4.3 Automation bias

Automation bias, the tendency to accept automated recommendations while disregarding contradictory information, was first described in aviation human-factors research (39) and later documented in clinical decision support (40). A plausible concern, not tested directly in the studies identified, is that generative models make it more likely, because fluent and assured language can convey more confidence than the underlying output warrants.

The clearest experimental estimate comes from the reader study of Dratsch and colleagues (21). Twenty-seven radiologists read 50 mammograms in two sets. In the first 10, which served to establish the credibility of the system, the suggested BI-RADS category was always correct; in the subsequent 40, it was incorrect for 12. When the suggestion was wrong, the proportion of correctly rated mammograms fell from 79.7% to 19.8% among inexperienced readers, from 81.3% to 24.8% among moderately experienced readers and from 82.3% to 45.5% among very experienced readers. Experience attenuated the effect but did not remove it.

The within-reader comparison and the stratification by experience give the study strong internal validity, but its figures cannot be generalized directly to practice (Supplementary Table S3). The system was simulated: readers saw suggestions they believed came from a newly developed AI product, so the study measures the response to purported algorithmic advice and not to a deployed tool. The error rate, 12 of 40 cases, far exceeds that of systems in clinical use, and the readings had no clinical consequences. The study therefore shows that radiologists are susceptible to incorrect advice under uncertainty, without providing an estimate of the size of the effect in routine screening.

Over-reliance of this kind is not specific to machines. In an experiment in which the same incorrect advice was attributed either to an AI system or to a human expert, physicians’ diagnostic accuracy was impaired to a similar extent (41), which suggests that the phenomenon reflects how clinicians take advice under uncertainty. An eye-tracking study examined a different kind of system error. Ten National Health Service breast-screening readers interpreted 60 examinations with and without AI prompts; on the cases the AI missed, median sensitivity fell from 71% unassisted to 39% with AI support, and gaze behavior changed only when the prompts were incorrect (22). The study is small and used a test set deliberately enriched for errors, and its result is treated here as a signal. It concerns cancers that the system failed to flag, as distinct from findings it flagged incorrectly.

One randomized trial bears on whether training protects against these effects (23). Forty-four physicians, all of whom had completed a 20 h AI-literacy program, were randomized to receive either unmodified LLM suggestions or suggestions containing deliberate, clinically significant errors in three of six vignettes, and could consult the model at their discretion. Physicians exposed to the erroneous suggestions reasoned less accurately. Because all participants had been trained, the trial had no untrained comparator. It shows that training did not prevent the effect in this cohort, but it neither estimates the effect of training nor shows that training is ineffective.

### 4.4 Skill retention after exposure to AI

If clinicians defer to AI when it is present, their performance may also change when it is absent. This possibility, usually termed deskilling, has long been discussed as an unintended consequence of medical AI (42, 43), but the search identified direct evidence in only one study.

Budzyń and colleagues conducted a retrospective observational study in four endoscopy centers in Poland that participated in the ACCEPT trial infrastructure (24). They compared the ADR during standard colonoscopies performed without AI in the three months before and the three months after CADe was introduced into routine practice. Across 1,443 unassisted procedures by 19 experienced endoscopists (795 before and 648 after), the ADR fell from 28.4% (226/795) to 22.4% (145/648), an absolute reduction of 6.0 percentage points (95% CI −10.5 to −1.6; P=.0089) and a relative reduction of about 21%. Exposure to AI remained associated with lower ADR in multivariable analysis (odds ratio 0.69, 95% CI 0.53–0.89).

The study measured a patient-relevant endpoint in four high-volume centers within an established research infrastructure (Supplementary Table S3). Its design, however, was a non-randomized pre–post comparison with observation periods of three months in a single country, so temporal trends, changes in case mix and seasonal variation remain possible explanations. The finding is an early signal that unassisted detection may decline after routine use of algorithmic support. It does not show that any loss of skill is lasting, that it occurs in other specialties, or that its mechanism is cognitive and not behavioral or organizational.

### 4.5 Undetected model failure and algorithmic bias

Automation bias and loss of skill concern the clinician’s response to a system. A different problem arises when the system itself performs poorly in ways that an individual clinician cannot observe.

The Epic Sepsis Model was deployed in hundreds of US hospitals before any independent evaluation. An external validation across 38,455 hospitalizations found an area under the receiver operating characteristic curve of 0.63 (95% CI 0.62–0.64), a sensitivity of 33%, a specificity of 83% and a positive predictive value of 12% (25). Two-thirds of sepsis episodes were missed and about 88% of alerts were false positives. The model had been in routine use without this level of performance being recognized; clinicians encounter alerts one patient at a time and are not in a position to judge calibration across a population.

Algorithmic bias can be equally hidden. Obermeyer and colleagues audited a commercial population-health algorithm, one of a class of tools that, according to industry estimates cited by the authors, are applied to about 200 million people in the United States each year (26). Using data from 49,618 primary care patients (6,079 Black and 43,539 White) at an academic hospital, they showed that the algorithm used past health expenditure as a proxy for illness. Because less is spent on Black patients with the same burden of illness, the algorithm systematically under-identified them for additional care. In the authors’ simulation, replacing cost with a direct measure of illness raised the proportion of Black patients identified for extra help from 17.7% to 46.5%; this is a modeled counterfactual and not the measured effect of a correction in practice. The model was accurate with respect to its training objective, and the flaw lay in the choice of label, a decision made upstream and invisible to its users (34).

Prospective evaluation of deployed systems appears to be uncommon. A scoping review of interpretable CDSS in high-risk pregnancy found that evaluations remain predominantly retrospective and that prospective studies of clinical impact are scarce (8).

### 4.6 Evidence from Latin America

To characterize the regional evidence, the search included dedicated queries in SciELO and LILACS in Spanish and Portuguese and a PubMed search restricted by Latin American geographical terms (Supplementary Table S2a). The regional records retrieved were mainly narrative overviews, ethical commentaries, surveys of digital-health readiness and retrospective machine-learning models developed and tested on historical local data. None met the eligibility criteria in Section 2.3, which require a comparison with unassisted clinicians or an outcome relevant to reasoning. The search therefore identified no primary trial, prospective human-in-the-loop evaluation or empirical study of automation bias, skill retention or diagnostic reasoning in clinical practice in Ecuador or the wider Andean region.

This result describes what the search found and does not show that no such research exists. Institutional repositories, grey literature, hospital quality-improvement reports, national thesis collections, conference proceedings and non-indexed regional journals were not searched, and any of them may contain relevant work. A regional scoping review with duplicate screening and coverage of grey literature would be the appropriate means of establishing the evidence base.

### 4.7 Summary of evidentiary strength

Table 2 lists the principal claims of this review with their evidentiary status, and Table 3 presents the study-level evidence on benefit and harm together. Demonstrated benefit is confined to perceptual screening and detection and rests mostly on intermediate endpoints; the one downstream endpoint available, interval cancer in MASAI, showed non-inferiority and not superiority. The null result for open-ended reasoning comes from one vignette-based trial with 50 physicians and six cases. It limits what can currently be claimed, but it does not show that LLM assistance cannot be made effective, because interface design, structured prompting and training were not tested as interventions. The evidence on harm concerns different mechanisms studied with different designs, and it is not combined here into a single estimate.

**Table 2:** Evidentiary status of the principal claims advanced in this review, classified according to Section 2.6.

Claim	Status	Principal basis
AI-supported reading increases cancer detection and reduces screen-reading workload in population mammography screening	[D]	Single large RCT, three protocol-defined analyses (16–18)
AI-supported reading yields a non-inferior interval-cancer rate and higher sensitivity in that program	[D]	Completed MASAI trial (18)
Real-time CADe increases adenoma detection rate during colonoscopy	[D]	Two RCTs, one multicenter (19, 35)
Access to an advanced LLM did not improve physician diagnostic reasoning scores on complex vignettes	[D]	Single RCT (20)
Interaction factors, rather than model capability, explain the gap between standalone and collaborative performance in open-ended reasoning	[H]	Interpretation of (20); mechanism not measured
Incorrect AI suggestions degrade reader accuracy at all experience levels	[S]	Single reader experiment with a simulated AI and an artificially high error rate (21)
AI false-negative prompts reduce reader sensitivity and alter visual search	[S]	Single paired eye-tracking reader study, enriched test set (22)
Physicians who completed AI-literacy training were still degraded by erroneous LLM advice	[S]	RCT without an untrained comparator (23)
Routine AI exposure is followed by reduced unassisted adenoma detection	[S]	Single multicenter observational pre–post study, 3-month window (24)
A deployed proprietary model can fail silently under independent external validation	[D]	External validation cohort (25)
Proxy-label choice can generate demographic disparity invisible to end users	[D]	Algorithmic audit (26)
Post-hoc explanations can raise trust without improving discrimination	[S]	Conceptual and empirical critique (44)
AI deployment increases defensive diagnostic testing	[H]	Mechanistic reasoning from liability doctrine (32); untested
Disagreement/override rates and unassisted competence audits merit evaluation as safety indicators	[N]	Author proposal; requires prospective validation
No eligible primary evidence on human–AI reasoning interaction in Latin America was identified by this search	[S]	Absence of eligible records in the present search only; see Section 4.6

Supplementary Table S4 maps each claim to its supporting record and to the manuscript section in which it appears.

**Table 3:** Primary studies anchoring this review, presenting benefit and harm evidence in a single frame, with CASP-based appraisal.

Domain & study	Design & sample	Principal quantitative finding	Methodological appraisal (CASP)	Evidence level
Screening mammography (16–18)	RCT; 105,934 randomized (80,033 at interim safety analysis)	Interim: 6.1 vs. 5.1 per 1,000 (ratio 1.2, 95% CI 1.0–1.5; P=.052); workload −44.3%. Full population: 6.4 vs. 5.0 per 1,000 (ratio 1.29, 95% CI 1.09–1.51; P=.0021); workload −44.2%. Completed: interval cancer 1.55 vs. 1.76 per 1,000 (ratio 0.88, 95% CI 0.65–1.18); sensitivity 80.5% vs. 73.8% (P=.031)	Population-based; prospective; single national program and single AI system; mortality and overdiagnosis not assessed	Level 1b (RCT)
Colonoscopy CADe (19)	Multicenter RCT; 685 patients	ADR 54.8% vs. 40.4% (RR 1.30, 95% CI 1.14–1.45); gain concentrated in adenomas ≤9 mm; withdrawal time unchanged	Adequate power for ADR; no long-term cancer outcome; industry-supplied equipment	Level 1b (RCT)
General diagnosis (20)	RCT; 50 attending/resident physicians, 6 cases	LLM access did not improve reasoning score (76% vs. 74%; adjusted difference 2 pp, 95% CI −4 to 8; P=.60); standalone LLM 92% (P=.03)	Validated reflective rubric; blinded grading; vignettes rather than live encounters; small sample	Level 2b (simulated RCT)
Mammography automation bias (21)	Prospective within-reader experiment; 27 readers; 10-case credibility set plus 40-case test set	With incorrect suggestions, correct BI-RADS assignment fell from 79.7% to 19.8% (inexperienced), 81.3% to 24.8% (moderate) and 82.3% to 45.5% (very experienced)	Stratified by experience; *simulated* AI (pseudo-prompts); 12 of 40 test cases erroneous, far above real-world failure rates; non-consequential setting	Level 3 (reader study)
Visual search (22)	Retrospective paired multireader eye-tracking study; 10 NHS readers, 60 enriched cases	On AI false-negative cases, median sensitivity fell from 71% (unassisted) to 39% (AI-assisted); fixation behavior altered only by incorrect prompts	Paired design with washout; objective gaze metrics; small reader sample; deliberately enriched error frequency	Level 3 (reader study)
AI-literacy training (23)	Single-blind RCT; 44 AI-trained physicians, 6 vignettes (errors seeded in 3)	Erroneous LLM advice degraded diagnostic reasoning relative to error-free advice among physicians who had completed 20 h of AI-literacy training	Randomized, prospectively registered; *no untrained comparator*, so the effect of training itself was not estimated; single country	Level 2b (simulated RCT)
Colonoscopy skill retention (24)	Retrospective multicenter pre–post; 1,443 unassisted procedures, 19 endoscopists	Unassisted ADR fell 28.4% (226/795) to 22.4% (145/648); −6.0 pp (95% CI −10.5 to −1.6; P=.0089); adjusted OR for AI exposure 0.69 (0.53–0.89)	Patient-relevant endpoint; multicenter; non-randomized pre–post; 3-month window; temporal and case-mix confounding not excluded	Level 3 (cohort)
Sepsis prediction (25)	External validation; 38,455 hospitalizations	Deployed commercial model AUC 0.63 (95% CI 0.62–0.64); sensitivity 33%, specificity 83%, PPV 12%	Large cohort; objective criteria; single health system network	Level 3 (validation cohort)
Population health equity (26)	Retrospective algorithmic audit; 49,618 patients (6,079 Black, 43,539 White)	Cost proxy produced racial disparity at equal risk score; in the authors’ simulation, relabelling raised Black patient enrolment in extra care from 17.7% to 46.5%	Rigorous econometric and counterfactual modeling; single institution; correction is simulated, not deployed	Level 3 (algorithmic audit)

Full matrices in Supplementary Table S3, which contains one entry per row of this table.

## 5 Discussion

### 5.1 Principal findings and interpretation

The studies reviewed support neither of the two common accounts of AI in diagnosis, that it reliably amplifies clinical reasoning or that it inevitably erodes it. What they show is narrower. Clinicians assisted by AI detected more cancers and adenomas in randomized trials of bounded perceptual tasks, whereas physicians given access to an LLM did not reason measurably better in the one randomized trial of open-ended diagnosis, even though the model outperformed them when used alone.

One interpretation that accommodates both results is that benefit depends on how the task, the form of the system’s output and the clinician’s reliance fit together. In mammography and colonoscopy the task is bounded, the output directs attention to a specific finding without offering a conclusion, and the clinician can check it at low cost by inspecting the flagged region. In open-ended reasoning the output is a diagnostic conclusion expressed in fluent language, and checking it requires much the same reasoning as reaching a conclusion independently. A model that is more accurate than its users may then add little to their performance, because they cannot readily tell when its advice should be accepted. This reading agrees with the explanations offered by the authors of the LLM trial (20) and with the uncalibrated pattern of reliance in Figure 1, but the trial did not test it, and it remains a hypothesis.

The studies of harm can be read in the same way, with the same caveat. When advice was wrong and could not easily be checked, readers at every level of experience tended to follow it (21). When the system failed to flag a cancer, readers detected it less often than they did unaided (22), which suggests that the absence of a prompt also shapes behavior and that a missing alert may be as consequential as an incorrect one. The fall in unassisted adenoma detection (24), if confirmed, would indicate that a prompt can come to substitute for the endoscopist’s own vigilance. Undetected model failure differs in kind, because it occurs at the level of the population and cannot be corrected by any individual act of verification.

For the evaluation of diagnostic AI, the implication is that standalone accuracy is a poor guide to clinical benefit and that assisted and unassisted performance, reliance and disagreement should be measured as primary outcomes. The evidence is also asymmetric. Benefit is supported by large trials with intermediate endpoints, whereas harm rests on small or design-limited studies. Trials are usually powered to detect benefit and seldom to detect harm, so this asymmetry may reflect how research has been designed more than the true balance of effects. The lack of strong evidence of harm should not be read as evidence of safety, and the interval-cancer result from MASAI is valuable for this reason, since downstream safety endpoints of this kind are rare.

### 5.2 Explainability as a safeguard

Explainable AI (XAI) is the usual technical response to opacity. Saliency maps, local attribution methods such as SHAP and LIME, and natural-language rationales are meant to let clinicians check the system’s reasoning and intercept its errors. This expectation is often not met. Post-hoc explanations are approximations computed after the prediction; they do not reproduce the model’s internal computation, they can change with small perturbations of the input, and different methods can yield contradictory explanations for the same prediction (44). A persuasive explanation may therefore increase trust without improving the clinician’s ability to distinguish correct from incorrect output, in which case it would reinforce the automation bias described in Section 4.3 instead of correcting it.

Interpretability remains relevant, provided that post-hoc rationales, which persuade, are distinguished from intrinsic interpretability, which reflects how the model actually works, as in generalized additive models, explicit uncertainty estimates and auditable provenance (10). For patient-facing and decision-support systems, a realist review of LLM safety guardrails likewise favors concrete safeguards, such as retrieval anchored to identified sources, explicit display of uncertainty and defined escalation pathways, over reliance on textual justification alone (13).

### 5.3 Liability and accountability

Malpractice law defines negligence as deviation from the customary standard of care. Medical AI unsettles that standard because adoption is uneven: its use is not yet customary, and failing to use it is not yet negligent (32). In this transitional situation, a clinician who follows an incorrect recommendation may be liable for misdiagnosis, whereas one who rejects a correct recommendation may be liable for disregarding available information.

These questions have begun to reach practice. In an opinion article published in September 2026, Sokol reports that NHS Resolution, which handles clinical negligence claims for the National Health Service in England, has received its first claims in which the use of AI in patient care is identified as a possible factor, and that the General Medical Council has received referrals concerning doctors’ misuse of AI systems (33). The source is a professional-ethics opinion and not peer-reviewed research. Its practical recommendation is nevertheless consistent with the human-factors evidence reviewed here: clinicians should be able to justify accepting or rejecting an algorithmic recommendation on grounds other than the fact that the system produced it, and should record that justification. Such documentation would make disagreement between clinician and system visible in the clinical record, which is a precondition for the monitoring proposed in Section 5.4.

Legal uncertainty may also encourage defensive practice, since clinicians could order additional tests to resolve ambiguous AI output. This prediction is plausible but untested, and it would require longitudinal health-economic study before it could be presented as an established effect.

### 5.4 Governance and candidate safety indicators

The sepsis and population-health cases show that calibration and fairness cannot be checked at the bedside. They have to be monitored at the level of the system, through local validation before deployment, performance reporting by subgroup and automated detection of distribution shift. The statutory and normative frameworks most relevant to this task are summarized in Table 4. The European provisions reflect Regulation (EU) 2024/1689 as amended by Regulation (EU) 2026/1744 of 8 July 2026 (the Digital Omnibus on AI), which was published in the *Official Journal of the European Union* on 24 July 2026 and entered into force on 27 July 2026 (45, 46); they were verified against the official text on 20 September 2026. The amendment revises the timetable in Article 113. Obligations for stand-alone high-risk systems listed in Annex III now apply from 2 December 2027 instead of 2 August 2026, and obligations for high-risk AI embedded as a safety component in products covered by the sectoral legislation of Annex I, the category that includes most AI-enabled medical devices under Regulation (EU) 2017/745, apply from 2 August 2028 instead of 2 August 2027. The transparency obligations of Article 50 took effect as originally scheduled on 2 August 2026. Because the timetable has already been amended once, the consolidated EUR-Lex text should be consulted before these dates are relied upon.

**Table 4:** Principal statutory and normative frameworks relevant to AI-supported clinical reasoning.

Framework	Statutory scope	Operational requirements for clinical reasoning
Regulation (EU) 2024/1689 (AI Act), as amended by Regulation (EU) 2026/1744 (45, 46)	European Union; AI Act in force since 1 August 2024; amending Regulation in force since 27 July 2026; legally binding, tiered application	Medical AI is high-risk under Annex I (devices under Regulation (EU) 2017/745) and Annex III; requires technical robustness, post-market surveillance, human oversight measures and data governance. Timetable after amendment of Art. 113: prohibited practices from 2 February 2025; general-purpose AI obligations from 2 August 2025; Article 50 transparency obligations from 2 August 2026; two further prohibitions and the shortened marking deadline for AI-generated or manipulated content from 2 December 2026; national regulatory sandboxes from 2 August 2027; Annex III stand-alone high-risk from 2 December 2027; Annex I embedded high-risk from 2 August 2028.
WHO Guidance on Large Multi-Modal Models (47)	Global non-binding normative guidance; published January 2024	Delineates ethical principles across developer, health system and clinician tiers; emphasizes transparency, human autonomy, validation across demographic strata and oversight of clinical reasoning applications.
PAHO Policy CD59/7 and Resolution CD59.R2 (48)	Region of the Americas; adopted 2021	Regional public health mandate for data science and AI; establishes ethical principles, digital health infrastructure targets and governance standards; does not impose point-of-care validation obligations on member states.

EU provisions reflect Regulation (EU) 2024/1689 as amended by Regulation (EU) 2026/1744, verified 20 September 2026.

These instruments require human oversight but tend to treat it as a procedural safeguard and not as a cognitive activity. A requirement for a “human in the loop” can be satisfied by countersigning a recommendation without examining it, and the trial of Qazi and colleagues indicates that training alone may not prevent uncritical acceptance (23). Oversight is more likely to be meaningful if it is measured. Two candidate indicators are proposed here for that purpose; both are the author’s proposals and would require prospective validation before they could serve as governance metrics.

The first is the rate at which clinicians override algorithmic recommendations. Its interpretation is not straightforward. A near-zero rate may indicate uncritical acceptance, but it may equally reflect an accurate model, a simple case mix, a well-tuned alert threshold or a workflow that leaves no opportunity to disagree; conversely, a high rate may reflect alert fatigue and not vigilance. Override rates should therefore be studied as exploratory indicators alongside clinical audit and should not be used as standalone performance measures. The documentation standard discussed in Section 5.3 (33) would allow them to be interpreted as well as counted.

The second indicator is periodic assessment of unassisted competence. In light of the colonoscopy signal (24), units that use AI for procedures or image interpretation could audit performance without AI at regular intervals to detect change from baseline. The appropriate sampling frequency, the minimum detectable difference and the influence of case-mix drift on such audits are unknown, so this proposal is at present a research question and not a ready standard.

### 5.5 Implications for Latin America

Health systems in Latin America, including Ecuador, adopt algorithmic tools under epidemiological and structural conditions that differ from those in which the tools were developed, and the search identified no eligible study of how clinicians in the region reason with them (Section 4.6). Even allowing for the limits of that search, the gap has practical consequences. Models are developed mostly with data from tertiary centers in North America, Western Europe and East Asia, whereas pre-test probabilities, clinical spectrum and nutritional and infectious comorbidity differ considerably in Andean and Amazonian populations. Because predictive values depend on prevalence, a model calibrated in one setting may plausibly produce unacceptable false-positive or false-negative rates in another, although this has not been measured for the tools in question. Where specialists are scarce and patient volumes high, the pressure to defer to automated recommendations is likely to be greatest, while the infrastructure needed to detect model failure is least developed. Moreover, although PAHO Resolution CD59.R2 establishes regional commitments on digital health (48), point-of-care safeguards such as mandatory local validation in silent mode or equity audits have largely not been legislated at the national level. Table 5 sets out a research agenda that is feasible with regional resources; its entries are the author’s proposals and not evidence-based recommendations.

**Table 5:** Priority research agenda for clinical AI evaluation in Latin American health systems.

Research question	Feasible study design	Primary clinical endpoint
Does an imported model retain diagnostic calibration?	Prospective silent-mode cohort validation before clinical deployment	Calibration slope, Brier score and stratified AUC across regional demographic strata
Does AI assistance alter unassisted diagnostic vigilance?	Interrupted time-series registry across endoscopy or radiology centers	Unassisted detection rate (e.g., unassisted ADR) before and after AI integration
Does early AI access alter trainee clinical reasoning?	Multi-site cluster-randomized educational trial in residency programs	Blinded diagnostic reasoning rubric score on unassisted complex vignettes
Does deployment alleviate or widen healthcare disparities?	Prospective cohort stratified by rurality, socioeconomic index and indigenous language	Time to confirmed diagnosis and specialist referral completion rates
What are baseline clinician override patterns?	Prospective audit of electronic health record alert interactions	Clinician-level override rate stratified by alert specificity and case complexity

All entries are author-proposed priorities [N], not evidence-based recommendations.

### 5.6 Implications for education and emerging systems

The findings also bear on training. AI literacy did not prevent automation bias in the one trial that tested trained physicians (23). Curricula may therefore need to move beyond general instruction about AI toward practices intended to preserve reasoning, such as forming a differential before consulting the system, verifying disagreement explicitly and practicing periodically without assistance (9). A related evaluation problem arises when LLMs are used as tutors, because fluent, Socratic-sounding dialogue can be mistaken for gains in reasoning (49). These are proposals, and their effect on diagnostic performance has not been evaluated.

Several current developments are likely to make these questions more pressing, although what follows extrapolates from observed trends and is not established. Agentic systems that break tasks into autonomous chains of retrieval, reasoning and tool use (14) would shift risk from isolated classification errors toward compounding multi-step errors whose origin is harder to trace. Conversational agents are increasingly used directly by patients for chronic-disease self-management and mental-health support (12, 13); in mental health they offer accessible interaction but lack the relational friction and boundaries of therapy and may reinforce users’ views uncritically (12). Algorithmic screening in retinal care extends access for underserved communities while altering the continuity of the relationship between patient and clinician (11). Each of these developments moves the system’s output away from bounded perceptual prompts and toward conclusions, the kind of output for which the present evidence shows no collaborative benefit.

### 5.7 Limitations

The most important limitations of this review concern selection. Sources were chosen purposively by a single author without independent duplicate screening, and no screening log was kept (Section 2.4). The corpus is a defensible but non-exhaustive selection, auditable through Supplementary Tables S2a, S2b; it is not a representative sample of the literature, and no claim is made about the completeness of the evidence base.

Several principal findings rest on few studies, notably a single observational study of skill retention (24) and a single vignette-based trial of open-ended reasoning (20), and the classification in Table 2 should constrain how they are used. As a narrative review reported under SANRA (6), the work involved no meta-analysis and no formal risk-of-bias assessment beyond the CASP appraisal of the nine anchor studies. The search did not include Embase, Web of Science, institutional repositories, thesis collections, preprint servers or unpublished hospital reports, so both the global and the regional pictures are incomplete to an unknown degree.

The evidence of benefit rests largely on intermediate endpoints: breast-cancer mortality and overdiagnosis remain unassessed in MASAI, and no colonoscopy trial cited here reports cancer incidence or mortality. The two principal reader studies of automation bias used deliberately high error rates, one of them with a simulated system, and therefore establish susceptibility and not realistic effect sizes. The regional finding reflects the absence of identified studies in one search, not evidence that such studies do not exist. Finally, the interpretation proposed in Section 5.1 is a hypothesis that has not been tested empirically.

## 6 Conclusions

The effect of AI on clinical reasoning cannot be inferred from the accuracy of a model tested alone. In the studies reviewed, AI assistance increased detection in mammography screening and colonoscopy, and in mammography it also reduced reading workload and yielded a non-inferior interval-cancer rate; effects on cancer mortality and overdiagnosis have not been reported. In open-ended diagnostic reasoning, the single randomized trial available found no improvement in physicians’ performance despite the higher score of the model alone, and it did not establish why.

The evidence that reliance on AI can impair performance is limited. Reader experiments show that radiologists are susceptible to incorrect and to missing AI prompts under experimental conditions. One trial shows that physicians trained in AI literacy were still misled by erroneous advice, one pre–post study reports lower unassisted adenoma detection after routine AI use, and one external validation and one audit show that deployed models can perform poorly or inequitably without this being recognized in use. These findings come from different designs and concern different mechanisms. They justify measurement and monitoring, but they do not establish the frequency, size or persistence of harm in routine practice.

Accordingly, the measures proposed here, namely monitoring of clinician–AI disagreement, periodic audit of unassisted performance and documentation of the reasons for accepting or rejecting algorithmic advice, are questions for prospective research and not ready standards. The search identified no eligible primary study from Latin America, and local validation of imported models, together with a regional scoping review with duplicate screening, is the necessary next step in that setting.
