# Supplementary material for: Augmentation or erosion? Artificial intelligence and the transformation of clinical reasoning: a critical narrative review
Source: Front Digit Health. 2026 Oct 21;8:1949323. doi: 10.3389/fdgth.2026.1949323 (PMC13732474; doi:10.3389/fdgth.2026.1949323)
Supplement: Supplementary file 1 [file Datasheet1.pdf]

Supplementary Material

Augmentation or Erosion? Artificial Intelligence and the Transformation of Clinical Reasoning: A Critical Narrative Review

José Daniel Sánchez Redrobán

October 2026

Note on scope. This Supplementary Material contains four items: the SANRA adherence statement (S1), the search documentation in two parts (S2a and S2b), the critical appraisal matrices (S3) and the claim-level evidentiary classification (S4). As stated in Methods, Section 2.4, of the main manuscript, no record-level screening log was kept, and no record counts or flow diagram are reported. The search is documented instead by an executable protocol (S2a) and by a classification of every cited source according to its route of identification (S2b), which can be checked against the manuscript's reference list. Section numbers in this document refer to the main manuscript.

Supplementary Table S1. SANRA adherence statement

Item-by-item adherence to the Scale for the Assessment of Narrative Review Articles (SANRA; Baethge, Goldbeck-Wood & Mertens, 2019). SANRA items are scored 0–2. The self-assessment below is declarative and is provided for editorial transparency, not as an external quality certification.

#	SANRA item	Score	Where and how addressed
1	Justification of the article's importance for the readership	2	Introduction: quantified burden of diagnostic error (Singh 2014; Newman-Toker 2024) and the distinction between standalone model accuracy and performance of the clinician–AI dyad.
2	Statement of concrete aims or formulation of questions	2	Introduction, final paragraph: two aims, each reported in Results (evidence on AI-assisted diagnostic performance, Sections 4.1–4.2; evidence on four risks of reliance, Sections 4.3–4.5), with the regional search in Section 4.6. The assessment of safeguards and the implications for governance, education and Latin America are addressed in the Discussion (Sections 5.2–5.6).
3	Description of the literature search	2	Methods 2.2 and Supplementary Table S2a: four named databases, complete Boolean strings, execution and refresh dates, date limits applied identically across databases, and a separately described regional stream. Methods 2.4 states explicitly what the search does *not* document (no record-level log, no counts).
4	Referencing	2	Every quantitative value is cited to the primary publication, not to a secondary review; the tracing procedure is described in Methods 2.4. All 48 references were checked against the publisher's record, PubMed or the official legal text. Source-level classification in S2b.

#	**SANRA item**	**Score**	**Where and how addressed**
5	Scientific reasoning	2	Methods 2.6 defines a four-level evidentiary classification ([D]/[S]/[H]/[N]), reported for each principal claim in Table 2 and in S4 and used in Table 1 and Figure 1; in the running text the status of a claim is stated in words. Findings (Section 4) are presented separately from interpretation and normative proposals (Section 5). Benefit and harm evidence are presented in a single comparative frame (Table 3).
6	Appropriate presentation of data	2	Effect estimates are reported with confidence intervals and P values as published, each analysis of a multi-report trial is presented separately (MASAI), and simulated or counterfactual results are labeled as such (Obermeyer relabeling). CASP appraisal of the nine anchor studies in S3.

Supplementary Table S2a. Database-specific search strings and execution dates

Initial execution 26 July 2026; refreshed 20 August 2026. The same date limits were applied in every database: no date restriction on foundational cognitive, methodological and normative literature; contemporary empirical retrieval restricted to 2015–2026. No yields are reported (Methods, Section 2.4).

Source	Query as executed
Scopus	`TITLE-ABS-KEY(("artificial intelligence" OR "machine learning" OR "deep learning" OR "large language model*" OR "generative AI" OR "clinical decision support*") AND ("clinical reasoning" OR "diagnostic error*" OR "diagnostic accuracy" OR "differential diagnosis" OR "automation bias" OR deskilling OR "human-AI interaction")) AND PUBYEAR > 2014 AND (LIMIT-TO(DOCTYPE,"ar") OR LIMIT-TO(DOCTYPE,"re"))`
PubMed/ MEDLINE	`("Artificial Intelligence"[Mesh] OR "Decision Support Systems, Clinical"[Mesh] OR "large language model*"[tiab] OR "generative AI"[tiab]) AND ("Clinical Reasoning"[Mesh] OR "Diagnostic Errors"[Mesh] OR "clinical reasoning"[tiab] OR "automation bias"[tiab] OR deskilling[tiab] OR "human-AI interaction"[tiab]) AND 2015:2026[dp]`
PubMed regional stream	The PubMed string above `AND ("Latin America"[Mesh] OR "South America"[Mesh] OR "Latin America*"[tiab] OR Ecuador[tiab] OR Andean[tiab] OR Peru[tiab] OR Colombia[tiab] OR Bolivia[tiab] OR Brazil[tiab])`. Reported as a separate stream; it is a subset of the PubMed yield and is not additive to it.
SciELO	`("inteligencia artificial" OR "inteligência artificial" OR "aprendizaje automático" OR "modelos de lenguaje") AND ("razonamiento clínico" OR "raciocínio clínico" OR "error diagnóstico" OR "erro diagnóstico" OR "apoyo a la toma de decisiones clínicas" OR "apoio à decisão clínica")`, all indexes, 2015–2026.
LILACS	`(tw:("inteligencia artificial" OR "inteligência artificial")) AND (tw:("razonamiento clínico" OR "raciocínio clínico" OR "toma de decisiones clínicas" OR "sesgo de automatización"))`, 2015–2026, Spanish/Portuguese/English.
Hand searching	Reference lists of all retrieved primary studies and of the secondary reviews listed in S2b; and back-tracing of the primary source of every quantitative value encountered in a secondary source.

Supplementary Table S2b. Record-level classification of every source cited

All 48 references cited in the manuscript, classified by how each entered the review. **DB** = retrieved by the database search described in S2a. **CC** = retrieved by citation chaining or by back-tracing a quantitative claim to its primary source. **OUT** = foundational, methodological or normative instrument cited outside the contemporary search (pre-2015 cognitive literature, reporting and appraisal instruments, statutory and intergovernmental texts). **PS** = identified after the search window closed and added during revision, cited as a normative source only. Every line below corresponds to exactly one entry in the manuscript's reference list.

Source (first author, year)	Route	Role in the review
Singh 2014	OUT	Burden of outpatient diagnostic error; foundational epidemiology.
Newman-Toker 2024	DB	Burden of serious harms from diagnostic error.
Croskerry 2009	OUT	Dual-process model of diagnostic reasoning.
Croskerry 2013	OUT	Cognitive bias and clinical decision making.
Norman 2017	CC	Relative contribution of bias and knowledge deficits to diagnostic error.
Schmidt 1990	OUT	Illness scripts; cognitive theory of expertise.
Cowan 2001	OUT	Working memory capacity.
Kahneman 2011	OUT	Dual-process theory; foundational.
Parasuraman 2010	OUT	Origin of the automation-bias construct (human factors).
Goddard 2012	OUT	Systematic review of automation bias in clinical decision support.
Gaube 2021	DB	Advice-taking susceptibility with human- vs AI-attributed advice.
Dratsch 2023	DB	**Anchor study**: automation bias, mammography reader experiment.
Taib 2026	DB	**Anchor study**: eye tracking; effect of AI false negatives on reader sensitivity.
Qazi 2026	DB	**Anchor study**: automation bias among AI-literacy-trained physicians (RCT).
Budzyń 2025	DB	**Anchor study**: unassisted adenoma detection before and after AI exposure.
Topaz 2026	DB	Commentary on deskilling; cited as normative framing, not as evidence.
Cabitza 2017	DB	Unintended consequences of machine learning in medicine.
Goh 2024	DB	**Anchor study**: RCT of LLM influence on diagnostic reasoning.
Yanagita 2024	CC	Expert assessment of LLM-generated illness scripts.
Obermeyer 2019	DB	**Anchor study**: algorithmic equity audit; proxy-label bias.
Wong 2021	DB	**Anchor study**: external validation of a deployed sepsis model.
Char 2018	CC	Ethical framing of upstream design choices.
Topol 2019	DB	Rationale for algorithmic assistance in diagnosis.
Ghassemi 2021	DB	Critique of post-hoc explainability.
Price 2019	DB	Liability doctrine for physicians using AI.

Source (first author, year)	Route	Role in the review
Sokol 2026	PS	Professional-ethics opinion (BMJ, 17 Sep 2026); first NHS Resolution claims and GMC referrals involving AI; cited as a normative source and explicitly identified as not peer reviewed. Published after the 20 August 2026 search refresh.
Mansoor 2026	DB	Signposting review: reasoning with LLMs in medicine.
Bali 2026	DB	Signposting review: CDSS in oral and maxillofacial radiology.
El Badisy 2026	DB	Signposting review: interpretable CDSS in high-risk pregnancy.
Tavares 2026	DB	Signposting review: educational frameworks for AI-enhanced diagnosis.
Zheng 2026	DB	Signposting review: faithful vs persuasive interpretability.
Hanhart 2026	DB	Signposting review: AI and the doctor–patient relationship in retinal care.
Cavalera 2026	DB	Signposting review: generative AI in mental health.
Zhao (Safety) 2026	DB	Signposting review: safety guardrails in patient-facing LLM systems.
Zhao (CRISPR) 2026	DB	Signposting review: agentic decision support.
EU Regulation 2024/1689	OUT	AI Act; primary legal text.
EU Regulation 2026/1744	OUT	Digital Omnibus on AI; amending regulation, primary legal text.
WHO 2024	OUT	Guidance on large multi-modal models; normative instrument.
PAHO 2021	OUT	Policy CD59/7 and Resolution CD59.R2; regional normative instrument.
Lång 2023 (MASAI)	DB	**Anchor study**, report 1 of 3: interim clinical safety analysis.
Hernström 2025 (MASAI)	CC	**Anchor study**, report 2 of 3: full randomized population.
Gommers 2026 (MASAI)	CC	**Anchor study**, report 3 of 3: interval cancer, sensitivity, specificity.
Repici 2020	CC	**Anchor study**: CADe colonoscopy RCT.
Wang 2019	CC	Second CADe colonoscopy RCT (single-center).
Abràmoff 2018	CC	Pivotal trial of autonomous diabetic retinopathy screening.
Martinez-Gutierrez 2023	CC	Cluster randomized trial of automated LVO detection and thrombectomy times.
Baethge 2019	OUT	SANRA reporting instrument.
CASP 2018	OUT	Critical appraisal checklists.

Reconciliation rule. The count of lines in this table equals the number of entries in the manuscript's reference list (48). No source appears in the manuscript that is absent here, and no source appears here that is absent from the manuscript.

Supplementary Table S3. CASP appraisal matrices for the nine anchor studies

Appraisal adapted from the CASP checklists for randomized trials, cohort studies and diagnostic studies. This table contains exactly nine entries, one per row of Table 3 of the manuscript. The MASAI trial is appraised once, as a single trial with three protocol-defined analyses.

Study	Design	Strengths	Limitations bounding inference
1. MASAI (Lång 2023; Hernström 2025; Gommers 2026)	Randomized, controlled, non-inferiority, single-blinded, population-based screening-accuracy trial	Clear question; population-based recruitment within a national program; prospective randomization; protocol-defined analyses reported separately; downstream interval-cancer endpoint reported at completion; specificity unchanged, so gains are not purchased with false positives	One country, one screening program, one commercial AI system; radiologists were aware of AI availability; interim detection ratio was not statistically significant and should not be cited as a positive detection result; breast-cancer mortality and overdiagnosis not assessed; transportability to settings with different prevalence, equipment and reader density untested
2. Repici 2020	Multicenter randomized trial, 685 patients	Randomized allocation; histopathology as reference standard; adequate power for ADR; withdrawal time unchanged, excluding a simple time-on-task explanation	Surrogate endpoint (ADR), not cancer incidence or mortality; equipment supplied by the manufacturer and authors report consultancy relationships; short observation period; gain concentrated in small adenomas of uncertain clinical significance
3. Goh 2024	Randomized clinical trial; 50 physicians, 6 vignettes	Validated diagnostic reasoning rubric; blinded expert grading; includes a standalone-model arm, which is what makes the human–AI gap visible	Vignettes, not live encounters; small physician sample and few cases, so the confidence interval remains compatible with modest benefit; no manipulation of prompting, interface or training, so the mechanism of the null result is unidentified; single-session exposure
4. Dratsch 2023	Prospective within-reader experiment; 27 readers; 10-case credibility set plus 40-case test set	Experience stratification with three defined tiers; standardized multi-case testing; credibility-establishing phase mirrors the trust formation of real deployment; large and consistent effect across tiers	The assisting system was *simulated*: readers responded to pseudo-prompts, not to a deployed product; 12 of 40 test cases erroneous, far above real-world failure rates; non-consequential reading environment; no patient outcomes; effect magnitude is not transferable to routine screening

Study	Design	Strengths	Limitations bounding inference
5. Taib 2026	Retrospective paired multireader eye-tracking study; 10 readers, 60 examinations	Paired within-reader design with a washout interval; objective gaze metrics rather than self-report; ground truth by histopathology or 3-year follow-up; isolates the specific risk of AI false negatives	Small reader sample from a single national screening program; test set deliberately enriched with false negatives and false positives, so prevalence-dependent measures are not generalizable; retrospective; single AI system
6. Qazi 2026	Single-blind randomized clinical trial; 44 AI-trained physicians, 6 vignettes with errors seeded in 3	Prospectively registered; randomized error placement; discretionary LLM consultation, which reflects realistic voluntary use; CONSORT-adherent reporting	*No untrained comparator*: every participant had completed the 20-hour training, so the trial cannot estimate the effect of training and does not show that training is ineffective; single country; vignette-based; small sample; supports a classification as signal [S], not as demonstrated [D]
7. Budzyń 2025	Retrospective multicenter observational pre–post; 1,443 unassisted procedures, 19 endoscopists	Patient-relevant endpoint (ADR) rather than a proxy; four centers within an established trial infrastructure; consistent direction after multivariable adjustment (OR 0.69)	Retrospective and non-randomized; 3-month windows before and after, insufficient for a durable skill claim; temporal trend, seasonal variation and case-mix drift not excluded; single country; experienced endoscopists only; mechanism (cognitive vs behavioral vs organizational) not identified
8. Wong 2021	External validation cohort; 38,455 hospitalizations	Large consecutive cohort; objective sepsis criteria; independent of the vendor; directly quantifies the discrepancy between marketed and observed performance	Single health system network; retrospective; model version and site-specific tuning may differ elsewhere; no direct measurement of clinician response to the alerts, so the downstream reasoning effect is inferred, not observed
9. Obermeyer 2019	Retrospective algorithmic audit; 49,618 patients (6,079 Black, 43,539 White)	Rigorous econometric and counterfactual modeling; identifies the defect at the level of label choice rather than model fitting; direct morbidity measures used as the comparator	Single academic institution; proprietary algorithm; Black and White patients only, precluding intersectional analysis; the 17.7%→46.5% figure is a *simulated* relabeling, not a measured effect of a deployed correction; the "200 million people" figure is an industry estimate of the scale of this class of tools, not the study sample

Supplementary Table S4. Claim-level evidentiary classification

Every principal claim classified in Table 2 of the manuscript, with its evidentiary status, its supporting record and the section in which it is reported. This table contains exactly fifteen entries, one per row of Table 2, and the wording of each claim is identical to that table. [D] Demonstrated in the cited setting; [S] Signal; [H] Hypothesis; [N] Normative proposal (definitions in Methods, Section 2.6).

Claim as stated in the manuscript	Status	Supporting record	Manuscript location
AI-supported reading increases cancer detection and reduces screen-reading workload in population mammography screening	[D]	Lång 2023; Hernström 2025; Gommers 2026	§4.1.1; §4.7, Tables 2–3
AI-supported reading yields a non-inferior interval-cancer rate and higher sensitivity in that program	[D]	Gommers 2026	§4.1.1; §4.7, Tables 2–3
Real-time CADe increases adenoma detection rate during colonoscopy	[D]	Repici 2020 (multicenter RCT); Wang 2019 (single-center RCT)	§4.1.2; §4.7, Tables 2–3
Access to an advanced LLM did not improve physician diagnostic reasoning scores on complex vignettes	[D]	Goh 2024	§4.2; §4.7, Tables 2–3
Interaction factors, rather than model capability, explain the gap between standalone and collaborative performance in open-ended reasoning	[H]	Interpretation of Goh 2024; mechanism not measured	§4.2 (authors' untested explanations); §5.1; Table 2
Incorrect AI suggestions degrade reader accuracy at all experience levels	[S]	Dratsch 2023 (simulated AI; artificially high error rate)	§4.3; §4.7, Tables 2–3
AI false-negative prompts reduce reader sensitivity and alter visual search	[S]	Taib 2026 (enriched test set)	§4.3; §4.7, Tables 2–3
Physicians who completed AI-literacy training were still degraded by erroneous LLM advice	[S]	Qazi 2026 (no untrained comparator)	§4.3; §4.7, Tables 2–3
Routine AI exposure is followed by reduced unassisted adenoma detection	[S]	Budzyń 2025 (pre–post, 3 months)	§4.4; §4.7, Tables 2–3
A deployed proprietary model can fail silently under independent external validation	[D]	Wong 2021	§4.5; §4.7, Tables 2–3
Proxy-label choice can generate demographic disparity invisible to end users	[D]	Obermeyer 2019 (correction simulated)	§4.5; §4.7, Tables 2–3
Post-hoc explanations can raise trust without improving discrimination	[S]	Ghassemi 2021	§5.2; Table 2
AI deployment increases defensive diagnostic testing	[H]	Mechanistic reasoning from Price 2019; untested	§5.3; Table 2

Claim	Status	Supporting record	Location
Disagreement/override rates and unassisted competence audits merit evaluation as safety indicators	[N]	Author proposal; documentation standard from Sokol 2026; requires prospective validation	§5.4; Table 2
No eligible primary evidence on human–AI reasoning interaction in Latin America was identified by this search	[S]	Absence of eligible records in the present search only; no count claimed	§4.6; §5.5; Table 2

Use of the labels. The bracketed labels appear in Tables 1 and 2 and in Figure 1 of the manuscript. In the running text the status of each claim is stated in words (for example, “is treated here as a signal” or “remains a hypothesis”). Descriptive statements of law (Table 4 of the manuscript), of published reporting instruments and of the content of the cited studies are not classified, because they report the record and are not inferential claims.
